# US homicide rates increase when resources are scarce and unequally distributed

**DOI:** 10.1017/ehs.2023.31

**Published:** 2023-12-11

**Authors:** Weston C. McCool, Brian F. Codding

**Affiliations:** 1 Department of Anthropology, University of Utah, Salt Lake City, UT 84102, USA; 2 Society, Water, and Climate Interdisciplinary Research Group, University of Utah, Salt Lake City, UT 84102, USA

**Keywords:** applied evolutionary anthropology, behavioural ecology, inequality, poverty, lethal violence

## Abstract

As homicide rates spike across the United States, researchers nominate diverse causes such as temperature, city greenness, structural racism, inequality, poverty and more. While variation in homicide rates clearly results from multiple causes, many correlation studies lack the systematic theory needed to identify the underlying factors that structure individual motivations. Building on pioneering work in evolutionary human sciences, we propose that when resources are unequally distributed, individuals may have incentives to undertake high-risk activities, including lethal violence, in order to secure material and social capital. Here we evaluate this theory by analysing federal data on homicide rates, poverty and income inequality across all 50 US states for the years 1990, 2000 and 2005–2020. Supporting predictions derived from evolutionary social sciences, we find that the interaction of poverty (scarcity) and inequality (unequal distribution) best explains variation in US homicide rates. Results suggest that the increase in homicide rates during the height of the COVID-19 pandemic are driven in part by these same underlying causes that structure homicide rates across the US over the last 30 years. We suggest that these results provide compelling evidence to expand strategies for reducing homicide rates by dismantling structures that generate and concentrate sustained poverty and economic inequality.

## Graphical abstract

**Figure:**
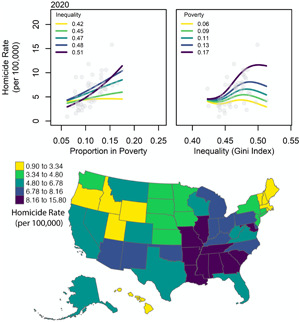

**Social media summary:** Homicide rates in the US increase when and where resources are scarce and unequally distributed.

## Introduction

Homicide rates across the US have been trending precipitously upward in the last few years (Lopez, 2022). Recent studies seeking to explain variation in homicide rates nominate diverse causes, including ambient temperature (Xu et al., 2020), city greenness (Sanciangco et al., 2021), firearm ownership (Studdert et al., 2022), firearm laws (Crifasi et al., 2018), structural racism (Unnever et al., 2021), income inequality (Daly, 2016), the interaction between absolute income and income inequality (Burraston et al., 2019; Gobaud et al., 2022), and more. While the homicide rate is clearly driven by multiple factors, many correlational studies lack the systematic theory needed to identify the underlying factors that structure individual motivations which may lead someone to kill another. Identifying causal factors is critical as interventions designed to reduce homicide rates need to target the underlying cause, rather than correlated effects. Here, we take an *a priori* approach by testing predictions derived from the evolutionary social sciences, rather than a purely empirical approach that attempts to infer causal links through model selection. Our analysis focuses on a set of predictor variables that have been hypothesised in evolutionary theoretical models to structure the underlying motivations for homicidal behaviour. While this approach may overlook proximate mechanisms and confounding variables, our goal is to draw attention to the ultimate factors influencing the individual payoffs for homicidal violence.

Researchers drawing on evolutionary theory propose the causal argument that absolute and relative resource scarcity structures the costs and benefits for violent interpersonal competition (Allen et al., 2016; Daly, 2016, 2023; Daly & Wilson, 1988; Daly et al., 2001; McCool et al., 2022). Specifically, when resources are unequally distributed, individuals with limited wealth and income may have incentives to undertake high-risk activities – including those that lead to lethal violence – in order to access material and social capital. In this sense homicide represents a ‘manifestation of the local level of competition for scarce resources’ (Daly, 2016: 2). This theory articulates with core predictions from life history theory, namely, that variation in resource availability and extrinsic mortality risk structure the costs and benefits of key life history trade-offs. In environments with high extrinsic mortality risk, fast life history strategies are favoured, which can include increased aggression, including homicide (Hackman & Hruschka, 2013; Wilson & Daly, 1997). Where poverty and inequality elevate extrinsic mortality risk, fast life history strategies may also promote future-discounting, where individuals place a premium on the present over the future (Wilson & Daly, 1997). These studies and others (e.g. Allen et al., 2016; de Courson et al., 2023; Daly & Krupp, 2022; Daly, 2023) develop a general evolutionary theory of lethal violence, which links material conditions necessary for survival and reproduction to the variable costs and benefits of homicide. Importantly, this theory recognises that (1) homicide cannot be explained with reference only to pathologies that produce maladaptive behaviours (Daly & Krupp, 2022; Hackman & Hruschka, 2013), (2) most homicide is, rather than a manifestation of mental illness, a complex, facultative capability sensitive to the conditional payoffs for competition over limited resources (Daly & Krupp, 2022) and (3) life history trade-offs may partially explain persistent cycles of killings (also termed enduring neighbourhood effects; de Courson et al., 2023), whereby homicide itself increases extrinsic mortality risk, which positively feeds back on life history strategies, and in addition, (4) we find it important to bring up the naturalistic fallacy, namely, that while homicide may be adaptive by providing Darwinian fitness benefits under certain conditions, this does *not* suggest that homicide is beneficial to society or morally appropriate.

This evolutionary theory, originally outlined by Daly and Wilson (1988), strengthens causal inferences that economists, sociologists and others have made for decades (see below), and can explain why resource scarcity and inequality so often correlate with harmful societal outcomes, including homicidal behaviour.

Building on the evolutionary theory outlined above, we recently showed that the link between resource limitations and violence is not unique to Western industrialised countries: generalised violence varies systematically across human societies as a function of relative and absolute resource availability (McCool et al., 2022). Specifically, our anthropological cross-cultural analysis shows that among delayed-return economies, a characteristic of agricultural as well as post-industrial societies, generalised violence representing homicide and warfare increases in environments where resources are scarce and heterogeneously distributed across space. In line with Daly et al.'s hypothesis, we argue that these results indicate that lethal violence is most often a manifestation of social competition for scarce, fitness-linked resources. In other words, the benefits for lethal violence and other risk-taking strategies should be sensitive to resource thresholds, below which social and economic standing – or survivorship in subsistence societies – is in jeopardy (de Courson et al., 2023). This theory thus predicts that homicide in delayed-return societies should peak when and where resources are scarce and/or unevenly distributed, and that the conditions that promote lethal violence may arise well before resource scarcity threatens basic survival. If the theory is correct, it may help explain the underlying causes of correlations between violence and many other factors including climate change (Hsiang et al., 2013), population pressure (Oka et al., 2017), agricultural productivity (Zhang et al., 2007) and more.

While the evolutionary social sciences provide a general theory grounded in Darwinian logic to elucidate why certain conditions promote homicide, there is an expansive literature in economics, psychology, political science and other sciences linking economic conditions and violence, much of which overlaps with evolutionarily motivated studies. While this literature is too vast to summarise in detail, we outline several important trends in the field pertaining to empirical approaches that establish links between economic conditions and homicide and studies which hypothesise explicit causal relationships. A more thorough review of non-evolutionary approaches can be found in Daly (2016, 2023).

A large amount of empirical research stems from the economics, sociology and political science literature, which has long proposed a positive relationship between various forms of inequality (e.g. ethnic inequality) and violent intergroup conflict (Cederman et al., 2011; Horowitz, 1985), including homicide (Aransiola et al., 2021; Burraston et al., 2019; Gobaud et al., 2022). Causal arguments are varied, but often propose violence to be a reaction to structurally imposed constraints on material and social capital leading to deprivation in certain communities (e.g. McDaniel et al., 2022; Daly, 2023; Vásquez et al., 2023). While this research programme has produced numerous empirical insights, studies often find conflicting results (e.g. Alesina et al., 2016; Cederman et al., 2011; Esteban & Ray, 2011; Huber & Mayoral, 2013) and some studies have called for a greater focus on theorising causal links between competing variables (Burke et al., 2015).

Prominent theories are too numerous to list here, but several influential models include the ‘social dominance’ hypothesis (Sidanius & Pratto, 2001), which proposes that societies create explicit hierarchies based on factors that privilege dominant groups, disadvantage subordinate groups and use physical force and ideological indoctrination to enforce norms of inequality, which in turn may spark violence within disadvantaged groups. Indeed, there is some evolutionary research suggesting ethnic dominance hinders contributions to public goods and may foment inter-ethnic conflict (Waring & Bell, 2013). The ‘social disorganisation theory’ suggests that local socio-economic conditions are more important than individual characteristics when explaining criminal activity and propose homicide to be a reaction to the breakdown of social cohesion in disadvantaged communities, which fosters feelings of resentment, frustration, hopelessness and alienation (Blau & Blau, 1982; Gobaud et al., 2022). These theories certainly differ from evolutionary models in their ultimate explanation of violent behaviour and focus more on the drivers of socially imposed constrains and the proximate phycological mechanisms that facilitate violent acts. Nonetheless, they articulate with evolutionary models in positing a causal relationship between resource deprivation and homicidal behaviour (Blume, 2021; Burraston et al., 2019; Gobaud et al., 2022; Vásquez et al., 2023).

Here we further evaluate this general evolutionary theory by systematically analysing data from the FBI Uniform Crime Reporting database on homicide rates, and the American Community Survey (ACS) on the proportion of households with income under the poverty level (proportion in poverty) and income inequality (Gini Index) across all 50 US states for the years 1990, 2000 and 2005–2020. Importantly, the 2020 data allow us to evaluate whether this general theory also partially accounts for the increase in violence during the COVID-19 pandemic. We also produce a novel analysis that examines how the effects of inequality vary across relatively rich vs. poor states and how the effects of poverty vary across relatively equal versus unequal states. These interactive effects should result from poverty and inequality targeting different aspects of resource availability. Poverty may push an entire population below a destitution threshold (de Courson et al., 2023), while inequality may drive resource scarcity in a subpopulation even as average holdings increase for the population at large (Daly, 2016). Thus, we expect homicide rates to peak when a meaningful proportion of a population is below a destitution threshold and existing resources are unequally distributed.

## Methods

**Figure 1.**
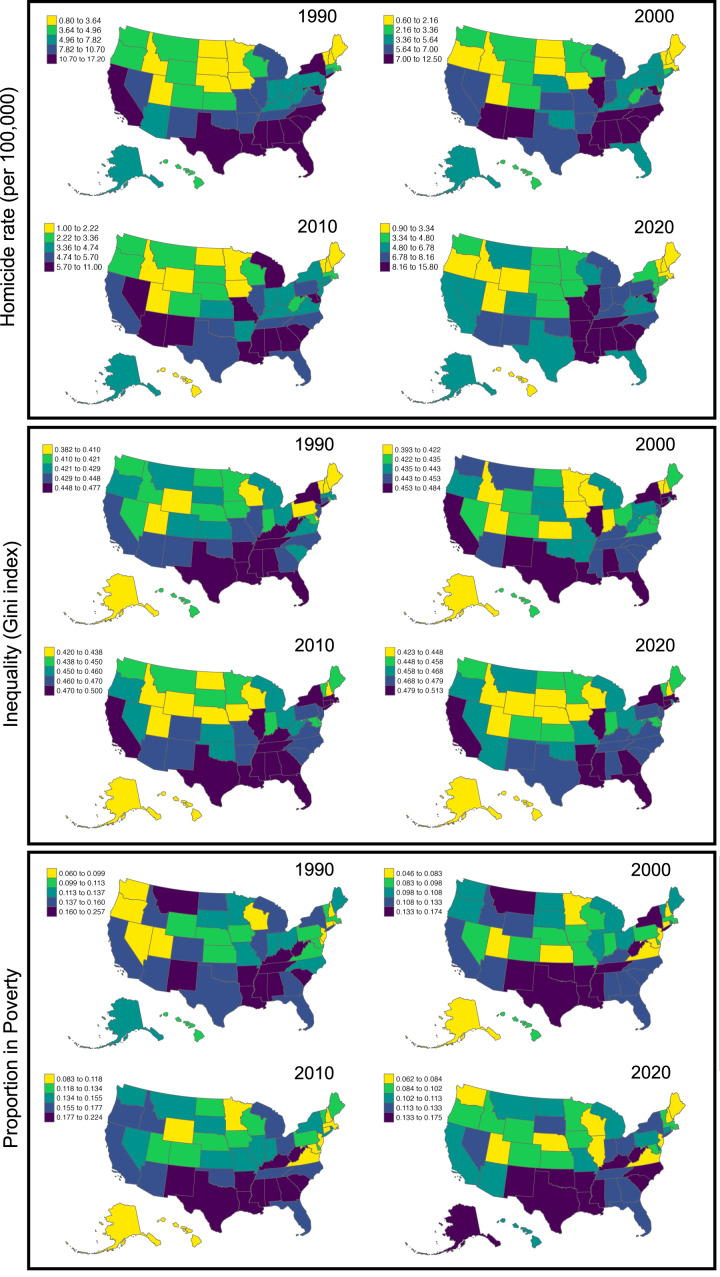
Distribution of homicide rates (per 100,000), Gini Index values and poverty proportions for each US state per decade in our study interval. Scales are not fixed to best highlight variation across time intervals.

Homicide, poverty and income inequality data were compiled for each US state in the years 1990, 2000 and 2005–2020. Homicide data derive from the FBI's Uniform Crime Reporting Program, Crime Data Explorer, Expanded Homicide Data. Data on inequality and the proportion of people estimated to be under the poverty level come from the ACS of the US Census Bureau (Figure 1). While Gini Index and Poverty data have been released for the year 2021, the current 2021 FBI Expanded Homicide Data include fewer homicides owing to an overall decrease in participation from agencies who are not yet reporting. Because of this systematic reporting bias, we do not include the 2021 data. Gini Index data are calculated from the Lorenz curve of household income within a state to measure income distribution from 0 to 1. The Gini Index is calculated from data in the US census population and can be found in the ACS and Supplementary Survey. While county- or zip-code-level data would provide higher resolution, these datasets are problematic. First, county-level data contain unreliable data for most US counties, and missing data for many rural counties. The National Center for Health Statistics Mortality Files considers homicide rate data to be unreliable when the standard error of the estimate is more than 20% of the estimate value and the measure value is outside the previous year's confidence interval. Second, much of the county-level data has a limited temporal extent, reducing diachronic variability when compared with state-level data. Third, zip-code data, which are derived from the National Violent Death Reporting System (Paulozzi et al., 2004), provide data only from participating states, which totalled 16 for most of the active data collection period. As such, state-level data are currently the most reliable and temporally extensive until higher resolution data are compiled and released. Prior to analysis, we evaluate the distribution of the response variable, and check for multicollinearity in our predictors using a linear model. More information about our data can be found in the SI Appendix. All data are provided in Dataset S1.

**Figure 2.**
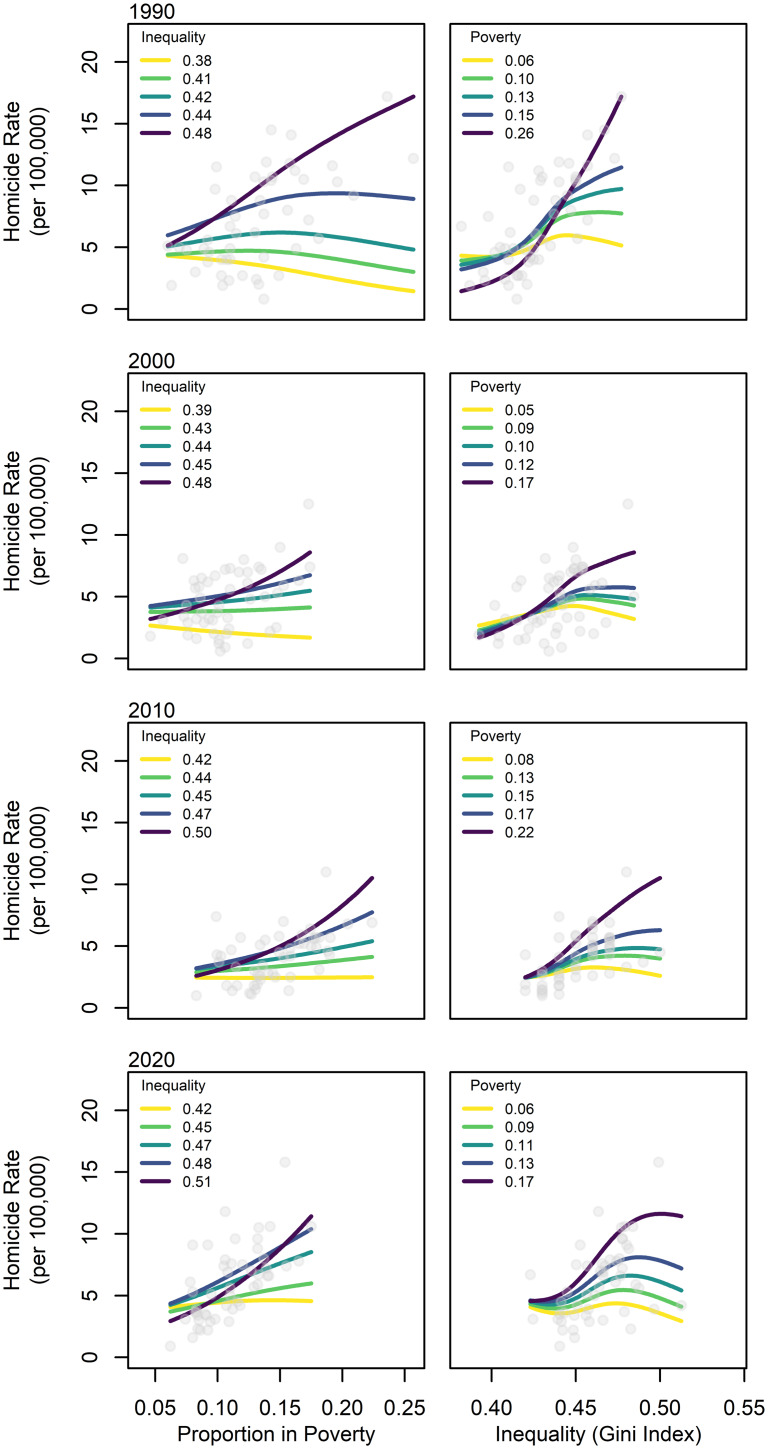
Partial response of homicide rate as a function of (left) the proportion of individuals in poverty for 0% (min), 25% (lower quartile), 50% (median), 75% (upper quartile) and 100% (max) quantiles of the Gini Index during the focal year, and of (right) the Gini Index for each quantile of the proportion of individuals in poverty during the focal year, both predicted for the years 1990, 2000, 2010 and 2020 (from top to bottom). Grey points show the observed value for each state in that focal year. Predicted model fits (lines) only cover the range of observed values.

We take a multi-model approach comparing seven increasingly complex statistical models that begin with a null model including only year as a random intercept, and end with a model that accounts for the interaction of poverty and inequality, and with a random intercept and factor-level smooths per year (see SI Appendix). We implement this approach using generalised additive models (Wood, 2017) that allow us to fit the predictor variables as linear parametric terms while also including random effects and non-linear terms. All models include year as a factor-level random intercept. This is because we have a ‘random’ sample of years for which data are reported, out of the population of all years of potentially available information on homicide rates, inequality and poverty. While we also have repeated observations per state, including both year and state as factor-level random effects would essentially account for all variation as the unique combination of each factor (state and year). As such, our data retain some non-independence; however, this is unavoidable. We attempt to partially account for this non-independence by fitting the trend for each year with random intercepts, and subsequently with random slopes and non-linear smooths. This allows the most complex models to fit trends in homicide across states as a function of the interaction between poverty and inequality, with a unique slope and intercept for each observation year. The two most complex models include smooths because the response may be non-linear; specifically, because payoffs for an individual to engage in risky behaviour should not accelerate indefinitely, but plateau or even decline at some threshold. Given the nature and distribution of the response variable, we specify a Poisson family and log link with quasi-likelihood estimation to relax assumptions and reduce potential overdispersion. Negative binomial models would produce comparable results. After fitting each model, we compare them in order of complexity using approximate hypothesis tests with an analysis of variance following standards outlined by Bolker et al. (2009). After selecting the ‘best’ model following the aforementioned procedure, we run diagnostics, including assessing standardised model residuals by evaluating their distribution, checking for overdispersion, assessing temporal autocorrelation averaged by year and comparing residuals by state. We then evaluate the results by examining model coefficients (Table 1) and plotting the partial response of predicted homicide rates as a function of poverty and inequality for the years 1990, 2000, 2010 and 2020 (Figure 2). We illustrate interactions between poverty and inequality by plotting the partial response of each across space for focal years for each of the 0% (minimum), 25% (lower quartile), 50% (median), 75% (upper quartile) and 100% (maximum) quantiles of the alternative variable. Predicted model fits are colour-coded using the viridis palette (Garnier et al., 2021) which is colour-blind friendly and translates well to greyscale. For example, the first panel in Figure 2 shows the predicted partial response of homicide to poverty across all states in 1990 for each quantile of the Gini coefficient. While our analysis is somewhat coarse grained, being limited to the available data at the state-year level, these patterns should hold at more fine-grained scales (Gobaud et al., 2022). Data and code required to reproduce all analyses are provided in the attached Dataset S1 and SI Appendix. All analyses are run in the R environment (R Core Team, 2022).

**Table 1.** Model coefficients for parametric and smoothed terms included in the best performing model. These include the estimate (*Est*.), standard error (SE), *t-*statistic (*t*) and *p*-value (*p*) for parametric terms, and estimated degrees of freedom (EDF), reference degrees of freedom (RDF), *F*-statistic (F) and *p* for smoothed terms

Parametric Terms	Est.	SE	T	p
(Intercept)	4.48	1.49	3.00	0.0028
Poverty	−51.60	11.84	−4.36	<0.0001
Inequality	−7.86	3.23	−2.43	0.0152
Poverty*Inequality	122.94	25.36	4.85	<0.0001

Smoothed Terms	EDF	RDF	F	p
s(Year)	16.17	17.00	15.96	<0.0001
s(Year, Poverty)	4.58	178.00	0.05	0.0088
s(Year, Inequality)	30.82	174.00	0.54	0.0002

## Results

From 2019 to 2020, homicide rates increased in 46 states, with only Alaska, Maine, New Hampshire and New Mexico showing declines (SI Appendix, Dataset S1). During this time, 38 states saw an increase in the proportion of households under the poverty line, and 35 states saw an increase in income inequality (Dataset S1).

Statistical models show that while poverty and inequality have independent positive effects on homicide, a model that includes their interaction plus random intercepts and factor-level smooths (equivalent to random slopes in a linear model) per year best explains variation in US homicide rates when compared with less complex models (*F* = 1.94, *p* = 0.0002). By extension, homicide rates are highest where poverty and inequality are highest. The model accounts for approximately 47% of the variation (measured in model deviance) in homicide rates (*r*^2^ = 0.46) with all terms contributing meaningfully (Table 1). As shown in Figure 1, from 1990 to 2020, predicted homicide rates peak in states where both inequality and poverty are highest.

Model residuals are centred on zero, and annually averaged residuals only harbour positive temporal autocorrelation up to one year. The residuals suggest that our model poorly fits 14 out of 900 cases (absolute standardised residuals > 3) by underpredicting homicide rates in several states with high rates, including Arkansas (2015, 2017, 2019), Louisiana (2009), Maryland (1990, 2005–2008, 2015–2017, 2019) and Nevada (2006). Such residual variation is not surprising given that this model leaves about 50% of the variation in homicide unaccounted for. Importantly, however, these outliers do not include observations during 2020, suggesting that the increase in homicide rates during the height of the COVID-19 pandemic is driven in part by these same underlying causes that structure homicides across the US over the last 30 years.

## Discussion

While our statistical methods are correlational, the underlying theory presents a causal argument proposing that individual decisions are more likely to result in situations leading to violence when and where resources are scarce and unequally distributed. Importantly, we do not argue that poverty and inequality are the *only* causes of homicide, nor do we contend that resource limitations will *determine* an individual's risk-preference. Rather, we argue that our results and those of related studies strongly suggest that relative and absolute resource availability exerts a strong influence on the costs and benefits of risk-taking behaviours, including lethal violence (Allen et al., 2016; de Courson et al., 2023; Daly, 2016; Daly et al., 2001; McCool et al., 2022). In other words, resource deprivation is a *sufficient* condition for rates of homicide to increase.

Multiple feedback mechanisms may also amplify violent competition. Prior research has shown evidence for a relationship between biased adult sex ratios and male violence, although whether more or fewer males relative to females increases or decreases male–male competition and homicide varies depending on the context (Schacht et al., 2014, 2022). Some evidence suggests that high male incarceration rates may lead to increased homicides (Messner and Sampson 1991). It is also possible that violent competition itself perpetuates homicidal behaviour by exacerbating overall resource shortfalls or fuelling rising inequality (Glowacki & Wrangham, 2013; Kelly, 2005). These ongoing processes may constitute often difficult to model enduring neighbourhood effects that, along with life-history trade-offs, can produce mismatches between homicide rates and current economic conditions.

While the current study focuses on resource limitations owing to poverty and inequality, this theory equally predicts that alternative mechanisms, such as climate change, should have important effects on lethal violence via their influence on resource availability. However, research shows that adverse climate conditions have disproportionately negative effects on populations with low resource holdings (IPCC, 2001, 2022), suggesting that climate effects will be strongly mediated by existing socio-economic conditions. This may help explain the underlying causes of climate-driven conflict observed globally (e.g. Hsang et al., 2013). This theory also suggests that adaptive strategies to reduce resource shortfalls will have mitigating effects on homicide and other risk-prone economic behaviours.

Our results also shed light on the ongoing debate concerning the relative importance of absolute poverty vs. inequality in promoting lethal violence (e.g. Daly & Krupp, 2022; Pridemore, 2011), suggesting that homicide rates are structured by the *interaction* of these two factors. Finally, as noted in previous studies (Daly & Krupp, 2022), unexplained residual variation in model results may be the product of spatial scale bias in variable measures. Estimates of homicide and inequality are at the level of states in our analysis and are often at the level of whole countries (Daly, 2023), while homicide is a manifestation of the interaction between *local* poverty and inequality. After all, individuals will surely use local cues of resource scarcity rather than state-wide or national markers. As such, future investigations will benefit from fine-grained data analysis to more fully capture the local fitness landscape, and studies reporting a lack of systemic links between inequality, poverty and homicide need to ensure that their results are not due to scaling error.

What do these results mean to efforts aimed at reducing homicide rates in the US? Our results indicate that resource deficiency may be structuring homicide rates throughout the last 30 years, including the 2020 increase. If this is true, interventions designed to reduce homicide rates need to target both the absolute and relative distribution of resources across the US. Put more directly, our results suggest that interventions that ignore poverty and inequality will probably fail to eliminate the core conditions that promote high-risk behaviours, including homicide. Importantly, this framework suggests that homicide rates disproportionately impact non-white communities owing to a long history of systemic racism towards racial and ethnic minority groups – especially Black communities (Schober et al., 2021) – that restrict individuals’ access to resources and opportunities (Daly, 2016; Yearby, 2018). Numerous studies have shown that across the US, in both contemporary and historic times, poverty and economic inequality are concentrated in minority communities due to ongoing legacies of systemic disadvantage (Bonilla-Silva, 2015; Daly, 2016; Schober et al., 2021; Unnever et al., 2021). It is thus no coincidence that these structurally imposed economic landscapes promote high-risk behaviours aimed at capturing key resources that can be attained by individuals from privileged backgrounds through safe, non-illicit means (Wilson & Daly, 1997). We suggest that these results, in line with arguments from Unnever et al. (2021), provide compelling evidence to expand strategies for reducing homicide rates by dismantling structures of systemic racism that generate and concentrate sustained poverty and economic inequality.

The goal of this paper is to contribute to the broader social science literature on homicide by providing an empirical assessment of resource conditions and homicide rates in the US, including the increase in homicides in 2020, and by articulating prominent social science theories within an evolutionary framework. Our results offer support for prior efforts that used smaller, less temporally extensive, datasets (e.g. Daly, 2016), and provides the most robust evaluation to-date of the interaction between poverty, inequality and homicide. Additional work is needed strengthen these conclusions. This includes the inclusion of other confounding variables, the application of causal modelling (Moscona et al. 2020) and approaches such as instrumental variable design (Hruschka & Henrich, 2013), and lastly, we call for additional homicide data to be compiled and released at higher spatial resolutions to more accurately capture local variation in homicide rates and economic conditions.

## Supporting information

McCool and Codding supplementary material 1 McCool and Codding supplementary material

McCool and Codding supplementary material 2 McCool and Codding supplementary material

## Data Availability

All data, code and materials used in the analysis are available in the supplementary materials.

## Research transparency and reproducibility

All data, code and materials used in the analysis are available in the supplementary materials.

## Supplementary material

To view supplementary material for this article, please visit https://doi.org/10.1017/ehs.2023.31

## References

[ref1] Alesina, A., Michalopoulos, S., & Papaioannou, E. (2016). Ethnic inequality. Journal of Political Economy, 124, 428–488. 27330223 10.1086/685300 PMC4907503

[ref2] Allen, M. W., Bettinger, R. L., Codding, B. F., Jones, T. L., & Schwitalla, A. W. (2016). Resource scarcity drives lethal aggression among prehistoric hunter–gatherers in central California. Proceedings of the National Academy of Sciences, 113, 12120–12125. 10.1073/pnas.1607996113 PMC5087046 27790997

[ref3] Aransiola, T. J., Ceccato, V., & Justus, M. (2021). The effect of absolute and relative deprivation on homicides in Brazil. Homicide Studies, 25, 361–386.

[ref4] Blau, J. R., & Blau, P. M. (1982). The cost of inequality: Metropolitan structure and violent crime. American Sociological Review, 47, 114–129.

[ref5] Blume, L. R. (2021). Narco Robin Hoods: Community support for illicit economies and violence in rural Central America. World Development, 143, 105464.

[ref6] Bolker, B. M., Brooks, M. E., Clark, C. J., Geange, S. W., Poulsen, J. R., Stevens, M. H. H., & White, J. S. S. (2009). Generalized linear mixed models: A practical guide for ecology and evolution. Trends in Ecology & Evolution, 24(3), 127–135. 19185386 10.1016/j.tree.2008.10.008

[ref7] Bonilla-Silva, E. (2015). The structure of racism in color-blind, ‘post-racial’ America. American Behavioral Scientist, 59, 1358–1376.

[ref8] Burke, M., Hsiang, S. M., & Miguel, E., (2015). Climate and conflict. Annual Review of Economics, 7, 577–617.

[ref9] Burraston, B., Watts, S. J., McCutcheon, J. C., & Province, K. (2019). Relative deprivation, absolute deprivation, and homicide: testing an interaction between income inequality and disadvantage. Homicide Studies, 23, 3–19.

[ref10] Cederman, L., Nils, B. W., & Gleditch, K. S. (2011). Horizontal inequalities and ethnonationalist civil war: A global comparison. American Political Science Review, 105, 478–495.

[ref11] Crifasi, C. K., Merrill-Francis, M., McCourt, A., Vernick, J. S., Wintemute, G. J., & Webster, D. W. (2018). Association between firearm laws and homicide in urban counties. Journal of Urban Health, 95, 383–390. 29785569 10.1007/s11524-018-0273-3 PMC5993701

[ref12] Daly, M. (2016). Killing the competition: Economic inequality and homicide. Routledge.

[ref13] Daly, M. (2023). Inequality, grievances, and the variability in homicide rates. Evolution and Human Behavior, 44, 296–304.

[ref14] Daly, M., & Krupp, D. B. (2022). Inequality, violence, and freedom. In L. E. Eppard & H. Giroux (Eds.), Inequality and freedom (pp. 117–125). Oxford University Press.

[ref15] Daly, M., Wilson, M. (1988). Homicide. Transaction.

[ref16] Daly, M., Wilson, M., & Vasdev, S. (2001). Income inequality and homicide rates in Canada and the United States. Canadian Journal of Criminology, 43, 219–236.

[ref17] De Courson, B., Frankenhuis, W. E., Nettle, D., & Van Gelder, J. L. (2023). Why is violence high and persistent in deprived communities? A formal model. Proceedings of the Royal Society B, 290, 20222095. 36809805 10.1098/rspb.2022.2095 PMC9943638

[ref18] Esteban, J., & Ray, D. (2011). Linking conflict to inequality and polarization. American Economic Review, 101, 1345–1374.

[ref19] Garnier, S., Ross, N., Rudis, R., Camargo, A. P., Sciaini, M., & Scherer, C. (2021). Rvision – Colorblind-Friendly Color Maps for R. R package version 0.6.2. https://www.rdocumentation.org/packages/Rvision/versions/0.6.0.

[ref20] Glowacki L., & Wrangham R. W. (2013). The role of rewards in motivating participation in simple warfare. Human Nature, 24, 444–460. 24008817 10.1007/s12110-013-9178-8

[ref21] Gobaud, A. N., Mehranbod, C. A., Dong, B., Dodington, J., & Morrison, C. N. (2022). Absolute versus relative socioeconomic disadvantage and homicide: a spatial ecological case–control study of US zip codes. Injury Epidemiology, 9, 1–9. 35216633 10.1186/s40621-022-00371-z PMC8876118

[ref22] Hackman, J., & Hruschka, D. (2013). Fast life histories, not pathogens, account for state-level variation in homicide, child maltreatment, and family ties in the US. Evolution and Human Behavior, 34, 118–124.

[ref23] Horowitz, Donald L. 1985. Ethnic Groups in Conflict. University of California Press.

[ref24] Hruschka, D. J., & Henrich, J. (2013). Institutions, parasites and the persistence of in-group preferences. PloS One, 8, e63642. 23704926 10.1371/journal.pone.0063642 PMC3660589

[ref25] Hsiang, S. M., Burke, M. & Miguel, E. (2013). Quantifying the influence of climate on human conflict. Science, 341, 1235367. 24031020 10.1126/science.1235367

[ref26] Huber, J. D., & Mayoral, L. (2013). Inequality, ethnicity and civil conflict. Working paper, Department of Political Science, Columbia University.

[ref27] IPCC (2001). Working Group II, Climate Change 2001: Impacts, adaptation and vulnerability, contribution of Working Group II to the third assessment report of the IPCC. Cambridge University Press.

[ref28] IPCC (2022). Working Group II, Climate Change 2022: Impacts, adaptation and vulnerability, contribution of Working Group II to the sixth assessment report of the IPCC. Cambridge University Press.

[ref29] Kelly R. C. (2005). The evolution of lethal intergroup violence. Proceedings of the National Academy of Sciences, 102, 15294–15298. 10.1073/pnas.0505955102 PMC1266108 16129826

[ref30] Lopez, G. (2022). Examining the Spike in Murders: The effects are felt unequally across the U.S. *New York Times.* nytimes.com/2022/01/18/briefing/crime-surge-homicides.

[ref31] McCool, W. C., Vernon, K. B., Yaworsky, P. M., & Codding, B. F. (2022). Subsistence strategy mediates ecological drivers of human violence. PLoS One, 17, e0268257. 35604917 10.1371/journal.pone.0268257 PMC9126380

[ref32] McDaniel, M., Ge, J., & Yuan, W. (2022). Social impacts of entrepreneurship: Does entrepreneurial ecosystem support reduce homicide? Journal of Business Venturing Insights, 17, e00315.

[ref33] Messner, S. F., & Sampson, R. J. (1991). The sex ratio, family disruption, and rates of violent crime: The paradox of demographic structure. Social Forces, 69, 693–713.

[ref34] Moscona, J., Nunn, N., & Robinson, J. A. (2020). Segmentary lineage organization and conflict in Sub-Saharan Africa. Econometrica, 88, 1999–2036.

[ref35] Oka, R. C., Kissel, M., Golitko, M., Sheridan, S. G., Kim, N. C. & Fuentes, A. (2017). Population is the main driver of war group size and conflict casualties. Proceedings of the National Academy of Sciences, 114, E11101–E11110. 10.1073/pnas.1713972114 PMC5748198 29229847

[ref36] Paulozzi, L. J., Mercy, J., Frazier, L., & Annest, J. L. (2004). CDC’s national violent death reporting system: Background and methodology. Injury Prevention, 10, 47–52. 14760027 10.1136/ip.2003.003434 PMC1756538

[ref37] Pridemore, W. A. (2011). Poverty matters: A reassessment of the inequality–homicide relationship in cross-national studies. The British Journal of Criminology, 51, 739–772.

[ref38] R Core Team (2022). R: A language and environment for statistical computing. R Foundation for Statistical Computing. https://www.R-project.org/.

[ref39] Sanciangco, J. C., Breetzke, G. D., Lin, Z., Wang, Y., Clevenger, K. A. & Pearson, A. L. (2021). The relationship between city ‘greenness’ and homicide in the US: Evidence over a 30-year period. Environment and Behavior, 2, 538–571.

[ref40] Schacht, R., Beissinger, S. R., Wedekind, C., Jennions, M. D., Geffroy, B., Liker, A., & Székely, T. (2022). Adult sex ratios: Causes of variation and implications for animal and human societies. Communications Biology, 5, 1273. 36402823 10.1038/s42003-022-04223-w PMC9675760

[ref41] Schacht, R., Rauch, K. L., & Mulder, M. B. (2014). Too many men: The violence problem? Trends in Ecology & Evolution, 29, 214–222. 24630906 10.1016/j.tree.2014.02.001

[ref42] Schober, D. J., Hunt, B. R., Benjamins, M. R., Saiyed, N. S., Silva, A., De Maio, F. G., & Homan, S., M. (2021). Homicide mortality inequities in the 30 biggest cities in the US. American Journal of Preventive Medicine, 60, 327–334. 33221143 10.1016/j.amepre.2020.09.008

[ref43] Sidanius, J., & Pratto, F. (2001). Social dominance: An intergroup theory of social hierarchy and oppression. Cambridge University Press.

[ref44] Studdert, D. M., Zhang, Y., Holsinger, E. E., Prince, L., Holsinger, A. F., Rodden, J. A., … Miller, M. (2022). Homicide deaths among adult cohabitants of handgun owners in California, 2004 to 2016: A cohort study. Annals of Internal Medicine, 175, 804–811. 35377715 10.7326/M21-3762

[ref45] Unnever, J. D., Stults, B. J., & Messner, S. F. (2021). Structural racism and criminal violence: An analysis of state-level variation in homicide. Race and Justice, doi: 10.1177/21533687211015287.

[ref46] Vásquez, A., Alvarado, R., Tillaguango, B., Işık, C., & Murshed, M. (2023). Impact of social and institutional indicators on the homicide rate in Ecuador: An analysis using advanced time series techniques. Social Indicators Research, 169, 1–22.

[ref47] Waring, T. M., & Bell, A. V. (2013). Ethnic dominance damages cooperation more than ethnic diversity: Results from multi-ethnic field experiments in India. Evolution and Human Behavior, 34, 398–404.

[ref48] Wilson, M., & Daly, M. (1997). Life expectancy, economic inequality, homicide, and reproductive timing in Chicago neighbourhoods. Bmj, 314, 1271. 9154035 10.1136/bmj.314.7089.1271 PMC2126620

[ref49] Wood, S. N. 2017. Generalized additive models: An introduction with R. CRC press.

[ref50] Xu, R., Xiong, X., Abramson, M. J., Li, S., & Guo, Y. (2020). Ambient temperature and intentional homicide: A multi-city case-crossover study in the US. Environment International, 143, 105992. 32738768 10.1016/j.envint.2020.105992

[ref51] Yearby, R. (2018). Racial disparities in health status and access to healthcare: The continuation of inequality in the United States due to structural racism. American Journal of Economics and Sociology, 77, 1113–1152.

[ref52] Zhang, D. D., Brecke, P., Lee, H. F., He, Y. Q. & Zhang, J. (2007). Global climate change, war, and population decline in recent human history. Proceedings of the National Academy of Sciences, 104, 19214–19219. 10.1073/pnas.0703073104 PMC2148270 18048343

