# Supplementary material for: US homicide rates increase when resources are scarce and unequally distributed
Source: Evol Hum Sci. 2023 Dec 11;6:e3. doi: 10.1017/ehs.2023.31 (PMC10955375; doi:10.1017/ehs.2023.31)
Supplement: McCool and Codding supplementary material 1 — McCool and Codding supplementary material [file S2513843X23000312sup001.pdf]

Supplementary Information for

Homicide rates in the United States are highest where resources are scarce and unequally distributed

Weston C. McCool* and Brian F. Codding*

- *weston.mccool@anthro.utah.edu* and *brian.codding@anthro.utah.edu*

This PDF file includes:

- Supplementary text
- Legend for Datasets S1
- SI References

Supplementary text

1 Introduction

This document is the supporting information (SI) appendix for the manuscript "Homicide rates in the United States are highest where resources are scarce and unequally distributed" by Weston C. McCool and Brian F. Codding. The document includes additional methods text and all the code required to reproduce the results and figures using the case data. All analyses are run in the R Environment for Statistical Computing (1).

2 Setup

Our analysis relies on generalized additive models from the `mgcv` library (2-6). Our figure uses colorblind safe palettes from the viridis library (7-8).

Load libraries:

```
library(mgcv)    #generalized additive models
library(viridis) #colorblind safe palette
```

3 Data

Homicide rate data come from the FBI's Uniform Crime Reporting Program, Crime Data Explorer, Expanded Homicide Data: https://crime-data-explorer.app.cloud.gov/pages/explorer/crime/shr

The proportion of people estimated to be under the poverty level data come from the American Community Survey (ACS, https://www.census.gov/programs-surveys/acs/) variable B17001 "Poverty Status in the Past 12 Months by Sex by Age" of the Census Bureau.

The Gini coefficient is derived from the Lorenz curve of household income within a state to measure income distribution from 0-1. The coefficient for each of the fifty states was calculated from data in the United States census population. Gini index data are derived from the Supplementary Survey and ACS for the years 2000-2020 and from Kahn et al. "State income inequality, household income, and maternal mental and physical health: cross sectional national survey" (2000) for 1990, which in-turn was derived from census data. https://www.census.gov/topics/income-poverty/income-inequality/about/metrics/gini-index.html

Data summary:

- year = record year
- state = reporting state
- homicide_rate = total number of homicides per 100,000 people

- gini_index = a measure of income distribution from 0 - 1
- poverty_prop = proportion of people with income under the poverty level

Read in the data:

```
df <- read.csv("Dataset_S1.csv")

head(df) #check the data

##   X year state homicide_rate gini_index poverty_prop
## 1 1 1990    AL          11.6     0.4570        0.192
## 2 2 2000    AL           7.4     0.4539        0.133
## 3 3 2005    AL           8.2     0.4728        0.169
## 4 4 2006    AL           8.3     0.4700        0.166
## 5 5 2007    AL           8.9     0.4700        0.169
## 6 6 2008    AL           7.5     0.4700        0.157
```

4 Exploratory data anlaysis

4.1 Distribution of the response variable

```
with(df,
     hist(homicide_rate,
          breaks = seq(0, round(max(homicide_rate),0)+1, by = 1),
          main = NA,
          xlab = "Homicide Rates")
  )
```

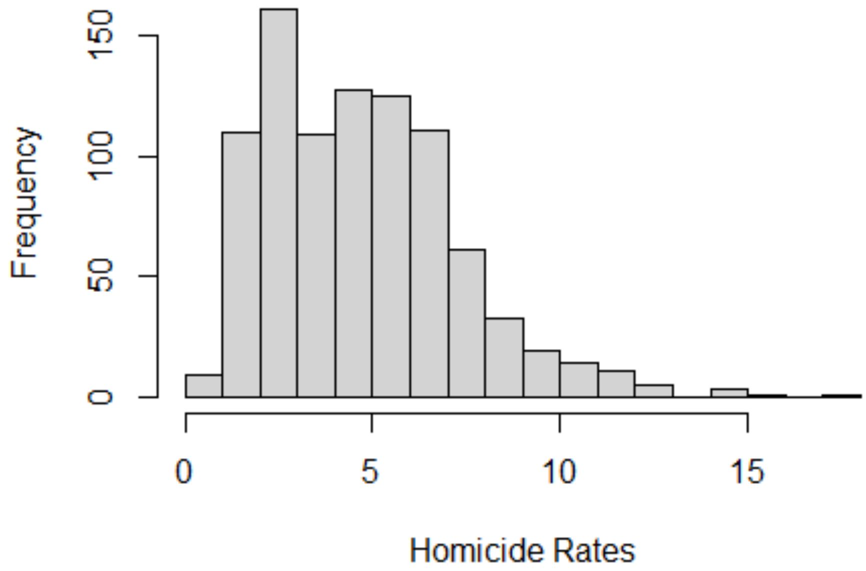

As reported in the main text, the response variable is highly skewed non-integer. We fit models specifying a Poisson family with quasi-likelihood estimation (“quasipoisson”).

4.2 Trends over time

Plot variation over time from 2000 to 2020 by state (grey) and US median (red):

```
state_name <- unique(df$state)

plot(NA,
     xlim = c(2000, 2020),
     ylim = c(0, max(df$homicide_rate)),
     ylab = "Homicide Rate (per 100,000",
     xlab = "Year"
     )

for(i in 1:length(state_name)){
with(subset(df, state == state_name[i]),
     lines(homicide_rate ~ year,
           lwd = 1,
           type = 'l',
           col = "grey"
           )
  )
}
```

```r
lines(aggregate(df$homicide_rate, by = list(df$year), FUN = median),
          lwd = 2,
          type = 'l',
          col = "red"
          )
```

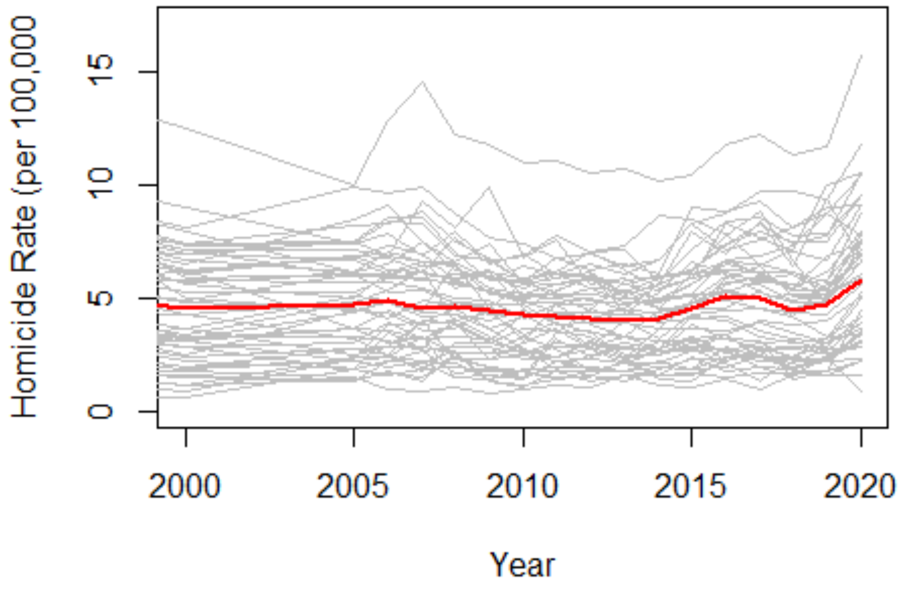

4.3 Variation by state

Which states had the highest homicide rates in 2020?

```r
head(subset(df[order(df$homicide_rate, decreasing = TRUE),], year == "2020"))

##       X year state homicide_rate gini_index poverty_prop
## 324 341 2020    LA          15.8     0.4991        0.154
## 450 467 2020    MO          11.8     0.4634        0.106
## 72   72 2020    AR          10.6     0.4792        0.142
## 432 449 2020    MS          10.6     0.4838        0.175
## 720 737 2020    SC          10.5     0.4770        0.133
## 18   18 2020    AL           9.6     0.4777        0.114
```

How many states show an increase from 2019 to 2020?

```r
state_increase <- with(df,
     homicide_rate[which(year == 2020)] > homicide_rate[which(year == 2019)]
)

length(state_name[which(state_increase==TRUE)])
```

```
## [1] 46
```

Which states show a decrease from 2019 to 2020?

```
state_name[which(state_increase==FALSE)]

## [1] "AK" "ME" "NH" "NM"
```

How many states showed an increase in poverty from 2019 to 2020?

```
state_poverty <- with(df,
     poverty_prop[which(year == 2020)] > poverty_prop[which(year == 2019)]
)

length(state_name[which(state_poverty==TRUE)]) #how many

## [1] 37
```

Which states did not show an increase in poverty?

```
state_name[which(state_poverty==FALSE)] #which not?

##  [1] "IL" "IA" "KS" "LA" "ME" "MS" "NE" "NY" "RI" "SC" "VT" "VA" "WI"
```

How many states showed an increase in inequality from 2019 to 2020?

```
state_inequality <- with(df,
     gini_index[which(year == 2020)] > gini_index[which(year == 2019)]
)

length(state_name[which(state_inequality==TRUE)]) #how many

## [1] 34
```

Which states did not show an increase in inequality?

```
state_name[which(state_inequality==FALSE)]

##  [1] "AK" "CT" "IN" "IA" "MD" "MS" "NV" "NH" "NM" "NY" "ND" "OH" "OK" "PA"
"UT"
## [16] "WA"
```

Which state-year has the highest homicide rate, poverty, and inequality?

```
head(df[order(df$homicide_rate, decreasing = TRUE), ])

##       X year state homicide_rate gini_index poverty_prop
## 307 324 1990    LA          17.2     0.4770        0.236
## 324 341 2020    LA          15.8     0.4991        0.154
## 311 328 2007    LA          14.6     0.4800        0.186
## 559 576 1990    NY          14.5     0.4670        0.143
## 757 774 1990    TX          14.1     0.4570        0.159
## 310 327 2006    LA          12.9     0.4800        0.190
```

```
head(df[order(df$poverty_prop, decreasing = TRUE), ])
```

```
##       X year state homicide_rate gini_index poverty_prop
## 415 432 1990    MS          12.2      0.473       0.2570
## 424 441 2012    MS           7.1      0.490       0.2415
## 425 442 2013    MS           7.3      0.480       0.2405
## 307 324 1990    LA          17.2      0.477       0.2360
## 421 438 2009    MS           6.6      0.470       0.2310
## 423 440 2011    MS           7.8      0.470       0.2255
```

```
head(df[order(df$gini_index, decreasing = TRUE), ])
```

```
##       X year state homicide_rate gini_index poverty_prop
## 129 129 2005    DE           4.4     0.5448       0.1030
## 573 590 2017    NY           2.8     0.5157       0.1408
## 575 592 2019    NY           2.9     0.5149       0.1250
## 571 588 2015    NY           3.1     0.5138       0.1540
## 574 591 2018    NY           2.9     0.5130       0.1110
## 572 589 2016    NY           3.2     0.5129       0.1473
```

4.4 Check for multicollinearity in predictors

Check for multicollinearity in predictor variables using a linear model and correlation coefficient.

```
lm.pov.ine <- lm(poverty_prop ~ gini_index, data = df)   #fit a linear model

lmr2.pov.ine <- round(
                  summary(                              #assign rounded r^2
to object
                    lm.pov.ine)$adj.r.squared,
                  2)

cor.pov.ine <- round(with(df,                           #with the data
                        cor(gini_index, poverty_prop)),#correlation coeffic
ient
                     2)                                 #round to 2 decimals
```

Print output:

```
lmr2.pov.ine #r^2
```

```
## [1] 0.18
```

```
cor.pov.ine  #r
```

```
## [1] 0.43
```

Plot output:

```
par(pty="s")                                           #square plot
```

```
with(df,                                                 #with the data frame
     plot(poverty_prop ~ gini_index,                     #plot poverty by Gini
          xlab = "Gini Coefficient",
          ylab = "Percent in Poverty"
          )
     )

abline(lm.pov.ine)                                       #add the lm fit

mtext(side = 3,                                          #add text reporting r
^2 and r
      adj = 1,
      cex = 0.75,
      text = bquote(r^2 ~ '=' ~ .(lmr2.pov.ine) ~ "," ~
                    r ~ '=' ~ .(cor.pov.ine))
      )
```

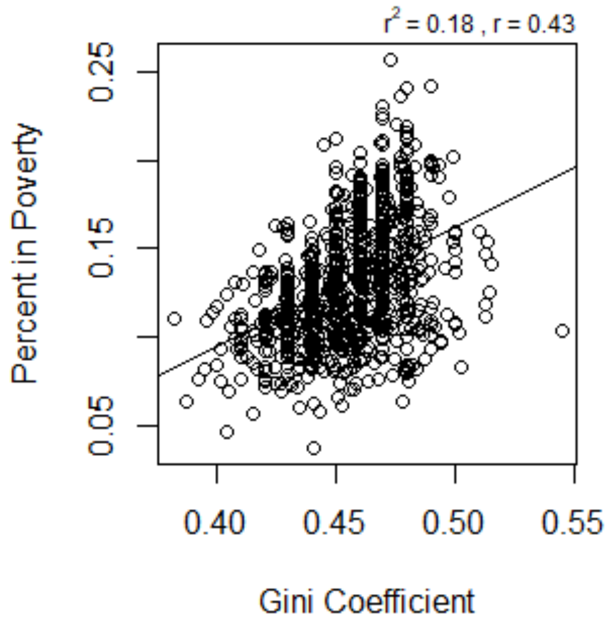

Following the “rule of thumb” supported by Dormann et al. (9), the reported level of multicollinearity should not bias model results.

5 Multimodel comparison

Following theory, we propose that absolute and relative income should influence decisions that may lead to homicide. The unit of analysis is the state-year. Here we construct seven increasingly complex models (below) using generalized additive models from the mgcv

library (2-6) which allows us to fit the predictor variables as linear parametric terms while also including random effects and non-linear terms. All models include year as a factor-level random intercept. This is because we have a “random” sample of years for which data are reported, out of the population of all years of potentially available information on homicide rates, inequality, and poverty. While we also have repeated observations per state, including both year and state as factor level random effects would essentially account for all variation as the unique combination of each factor (state and year). As such, our data retain some non-independence, however, this is unavoidable. Our attempt to partially account for this non-independence by fitting the trend for each year with random intercepts, and subsequently with random slopes and non-linear smooths. We include smooths because the response may be non-linear as pay-offs to risky behavior should plateau at some threshold. Given the nature and distribution of the response variable, we specify a Poisson family and log link with quasi-likelihood estimation to relax assumptions and reduce potential overdispersion. Negative binomial models would produce comparable results. After fitting each model, we compare them in order of complexity using approximate hypothesis tests with the `anova.gam` function. After selecting the “best” model, we run diagnostics, including assessing standardized model residuals by evaluating their distribution, checking for overdispersion, assessing temporal autocorrelation averaged by year, and comparing residuals by state. We then evaluate the results by examining model coefficients and plotting the partial response of predicted homicide rates as a function of poverty and inequality for the years 1990, 2000, 2010, and 2020.

Models:

- null model with only year as random intercept
- poverty only model with year as random intercept
- inequality only model with year as random intercept
- additive model with both poverty and inequality as parametric terms (linear) and year as random intercept
- interaction model with poverty and inequality as parametric terms and year as random intercept
- interaction model with poverty and inequality as parametric terms and year as random intercept and slope
- interaction model with poverty and inequality as parametric terms and year as random intercept and factor smooth (non-linear) interaction

Fit the models:

```
df$year <- as.factor(df$year)        #set year as a factor level

#null model with only year as a random intercept
nul.m <- gam(homicide_rate ~         #response: homicide rate
                s(year, bs = "re"),  #year as a random intercept
             family = quasipoisson,  #Poisson family with quasi-likelihood es
timation
             data = df               #with the data frame
              )
```

```
#poverty model + random intercept
pov.m <- gam(homicide_rate ~          #response: homicide rate
                poverty_prop +        #proportion living in poverty
                s(year, bs = "re"),   #year as a random intercept
             family = quasipoisson,   #Poisson family with quasi-likelihood es
timation
             data = df                #with the data frame
              )

#inequality model + random intercept
ine.m <- gam(homicide_rate ~          #response: homicide rate
                gini_index +          #Gini index
                s(year, bs = "re"),   #year as a random intercept
             family = quasipoisson,   #Poisson family with quasi-likelihood es
timation
             data = df                #with the data frame
              )

#poverty and inequality + random intercept
add.m <- gam(homicide_rate ~          #response: homicide rate
                poverty_prop +        #proportion living in poverty
                gini_index +          #Gini index
                s(year, bs = "re"),   #year as a random intercept
             family = quasipoisson,   #Poisson family with quasi-likelihood es
timation
             data = df                #with the data frame
              )

#poverty interacting with inequality + random intercept
int.m <- gam(homicide_rate ~          #response: homicide rate
                poverty_prop *        #proportion living in poverty interactio
n with
                gini_index +          #Gini index
                s(year, bs = "re"),   #year as random intercept
             family = quasipoisson,   #Poisson family with quasi-likelihood es
timation
             data = df                #with the data frame
              )

#poverty interacting with inequality + random intercept + slopes
int.b <- gam(homicide_rate ~          #response: homicide rate
                poverty_prop *        #proportion living in poverty interactio
n with
                gini_index +          #Gini index
                s(year, bs = "re") + #year as a random intercept
                s(year, poverty_prop,#with random slope for poverty
                  bs = "re")+
                s(year, gini_index,   #and random slope for inequality
```

```
                     bs = "re"),
                family = quasipoisson,  #Poisson family with quasi-likelihood es
timation
                data = df               #with the data frame
                 )

#poverty interacting with inequality + random intercept + smooths
int.s <- gam(homicide_rate ~            #response: homicide rate
                poverty_prop *          #proportion living in poverty interactio
n with
                gini_index +            #Gini index
                s(year, bs = "re") + #year as a random intercept
                s(year, poverty_prop,#with random smooth for poverty
                  bs = "fs")+
                s(year, gini_index,  #and random smooth for inequality
                  bs = "fs"),
                family = quasipoisson,  #Poisson family with quasi-likelihood es
timation
                data = df               #with the data frame
                 )
```

Compare the models:

```
anova.gam(nul.m,      #null model
          pov.m,      #poverty
          ine.m,      #inequality
          add.m,      #additive
          int.m,      #interaction
          int.b,      #interaction with random slope per year
          int.s,      #interaction with smooth factor interaction
          test = "F") #compare w/ F-test following R Core Team (2021) documen
tation stats::family

## Analysis of Deviance Table
##
## Model 1: homicide_rate ~ s(year, bs = "re")
## Model 2: homicide_rate ~ poverty_prop + s(year, bs = "re")
## Model 3: homicide_rate ~ gini_index + s(year, bs = "re")
## Model 4: homicide_rate ~ poverty_prop + gini_index + s(year, bs = "re")
## Model 5: homicide_rate ~ poverty_prop * gini_index + s(year, bs = "re")
## Model 6: homicide_rate ~ poverty_prop * gini_index + s(year, bs = "re") +
##     s(year, poverty_prop, bs = "re") + s(year, gini_index, bs = "re")
## Model 7: homicide_rate ~ poverty_prop * gini_index + s(year, bs = "re") +
##     s(year, poverty_prop, bs = "fs") + s(year, gini_index, bs = "fs")
##   Resid. Df Resid. Dev         Df Deviance         F    Pr(>F)
## 1    883.31    1088.12
## 2    881.07     746.51  2.2454995   341.62  195.8940 < 2.2e-16 ***
## 3    881.13     844.10 -0.0601301   -97.59 2089.8972 < 2.2e-16 ***
## 4    880.07     698.31  1.0556138   145.78  177.8285 < 2.2e-16 ***
## 5    879.07     694.83  0.9999905     3.48    4.4835 0.0345218 *
```

```
## 6    879.06     693.43  0.0051404     1.40  351.3644 0.0006692 ***
## 7    828.18     616.97 50.8818028    76.46    1.9350 0.0001446 ***
## ---
## Signif. codes:  0 '***' 0.001 '**' 0.01 '*' 0.05 '.' 0.1 ' ' 1

b.mod <- int.s        #assign "best" model to object
```

As reported in the main text, the interaction model with smoothed terms by year is a significant improvement on all less complex models.

6 Model diagnostics

6.1 Standardized residuals

```
b.mod.resid <- residuals(b.mod, type = "scaled.pearson")

     hist(b.mod.resid,
          main = NA,
          breaks = 100,
          xlim = c(-15, 15),
          xlab = "residuals\n(scaled Pearson)")
```

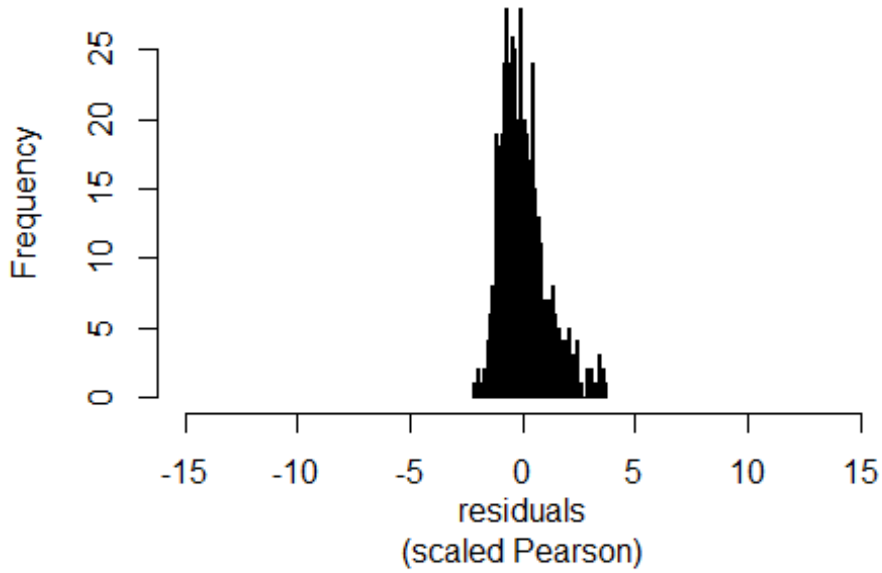

As reported in the main text, standardized residuals are centered on zero, but the model under-predicts several outlier cases (defined as scaled residual greater or less than 3).

These are:

```
df[which(b.mod.resid >= 3 | b.mod.resid <= -3), 1:3] #select state-year with outlier residuals greater than 3 or less than -3

##       X year state
## 31   31 2015    AK
## 33   33 2017    AK
## 35   35 2019    AK
## 313 330 2009    LA
## 343 360 1990    MD
## 345 362 2005    MD
## 346 363 2006    MD
## 347 364 2007    MD
## 348 365 2008    MD
## 355 372 2015    MD
## 356 373 2016    MD
## 357 374 2017    MD
## 359 376 2019    MD
## 490 507 2006    NV
```

Residuals for 2020:

```
     hist(b.mod.resid[which(df$year == "2020")],
          main = NA,
          breaks = 100,
          xlim = c(-15, 15),
          xlab = "residuals\n(scaled Pearson)")
```

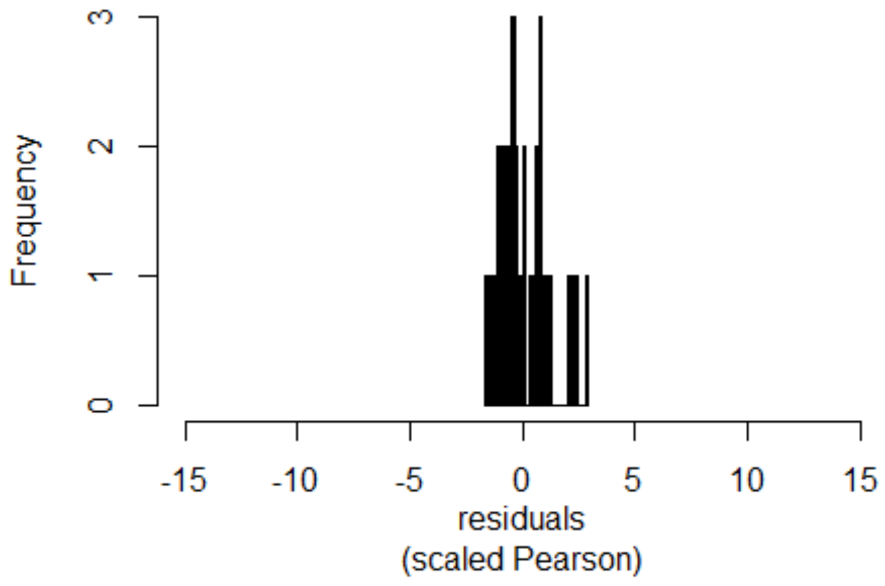

6.2 Overdispersion

Check for overdispersion in the residuals:

```
sum(residuals(b.mod, type = "pearson")^2) / df.residual(b.mod)

## [1] 0.7708686
```

6.3 Temporal autocorrelation

As there are multiple annual observations per state, we examine mean residual temporal autocorrelation per year:

```
b.mod.resid.mean <- aggregate(b.mod.resid, by = list(df$year), FUN = mean) #m
ean residual per year

b.mod.resid.mean <- b.mod.resid.mean[3:18,]#drop 1990 and 2000 given interrup
tions in time series

acf(b.mod.resid.mean$x, main = NA) #mean residual per year from 2005 to 2020
```

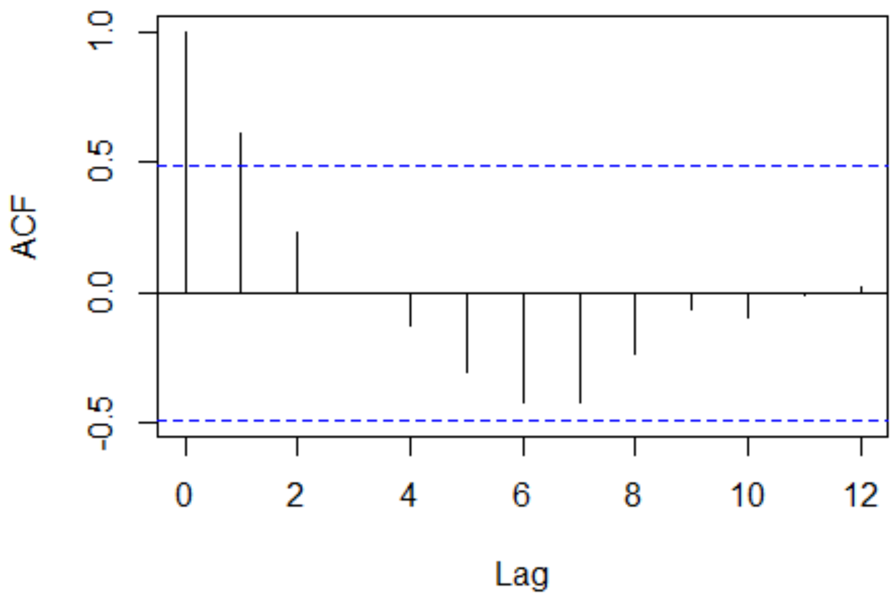

As reported in the main text, there is only meaningful averaged autocorrelation up to one year.

6.4 State-level variation in residuals

Examine median residuals by state:

```r
b.mod.resid.median <- aggregate(b.mod.resid, by = list(df$state), FUN = media
n)

par(pty = "s")

plot(b.mod.resid.median$x, 1:50,  #plot blank range of residuals
     yaxt = "n",
     pch = NA,
     xlab = "residuals",
     ylab = NA)

text(b.mod.resid.median$x, 1:50,  #add state labels
     labels = paste0(b.mod.resid.median$Group.1),
     cex = 0.75)

abline(v = 0, col = 'grey')       #add reference line
```

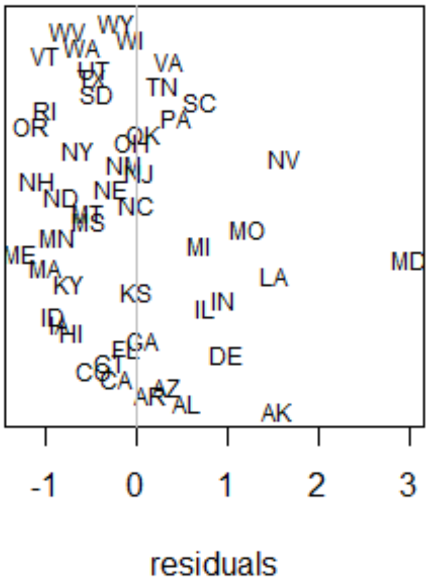

7 Model results

7.1 Model summary

Print the model summary:

```r
summary(b.mod)
```

```
##
## Family: quasipoisson
## Link function: log
##
## Formula:
## homicide_rate ~ poverty_prop * gini_index + s(year, bs = "re") +
##     s(year, poverty_prop, bs = "fs") + s(year, gini_index, bs = "fs")
##
## Parametric coefficients:
##                          Estimate Std. Error t value Pr(>|t|)
## (Intercept)                 4.478      1.492   3.001  0.00277 **
## poverty_prop              -51.600     11.840  -4.358 1.47e-05 ***
## gini_index                 -7.860      3.231  -2.433  0.01519 *
## poverty_prop:gini_index   122.936     25.362   4.847 1.49e-06 ***
## ---
## Signif. codes:  0 '***' 0.001 '**' 0.01 '*' 0.05 '.' 0.1 ' ' 1
##
## Approximate significance of smooth terms:
##                          edf Ref.df      F  p-value
## s(year)               16.172     17 15.962  < 2e-16 ***
## s(year,poverty_prop)   4.584    178  0.053 0.008836 **
## s(year,gini_index)    30.823    174  0.539 0.000239 ***
## ---
## Signif. codes:  0 '***' 0.001 '**' 0.01 '*' 0.05 '.' 0.1 ' ' 1
##
## R-sq.(adj) =  0.462   Deviance explained = 47.3%
## GCV = 0.77873  Scale est. = 0.77662   n = 900
```

7.2 Partial response

Isolate the inverse link of the model for transforming predicted values into response:

```
i.link <- family(b.mod)$linkinv #inverse link function
```

Set the number of values to predict:

```
n.int <- 100                          #number of values to predict across ran
ge

year_seq <- c(1990, 2000, 2010, 2020) #years to predict

n_levs <- 5                           #number of levels (quantiles)
```

Plot the partial responses for homicide rate as a function of the proportion of individuals in poverty for each quantile of the Gini index, and as a function of the Gini index for each quantile of the proportion of individuals in poverty, predicting only across the range of observed state values for that focal year.

```
#png("McCoolCodding_Fig1.png", height = 7.5, width = 4, units = "in", res = 1
200) #write file
```

```
#plot
par(pty = "s", mfrow = c(4,2), mar = c(0, 1, 0, 0), oma = c(2,4.5,1,1))#squar
e two panel plot

for (j in 1:length(year_seq)) {

#left panel

plot(NA,                                                                #blank
plot
     xlim = c(min(df$poverty_prop), max(df$poverty_prop)),             #range
of poverty values
     ylim = c(0 , max(df$homicide_rate)+5),                            #range
of homicide values + 5
     xlab = "",
     xaxt = "n",
     ylab = "Homicide Rate\n(per 100,000)",
     xpd = NA
  )

#add lines
for(i in 1:n_levs){                                                     #loop
to plot for each quantile
ine.pred.lev <- with(subset(df, year == year_seq[j]),
                            quantile(gini_index)                       #quant
ile levels of observed Gini in focal year
                     )

pov.pred.seq <- with(subset(df, year == year_seq[j]),                  #seque
nce to predict across range of poverty for focal year
                     seq(min(poverty_prop),
                         max(poverty_prop),
                         length.out = n.int)
                     )

pred.hom.pov <- data.frame(poverty_prop = pov.pred.seq,                #new d
ata across sequence of poverty
                           gini_index = rep(ine.pred.lev[i], n.int),   #for e
ach quantile of Gini
                           year = rep(year_seq[j], n.int)              #for f
ocal year
                           )

pred.hom <- predict(b.mod, newdata = pred.hom.pov)                     #predi
ct values


lines(i.link(pred.hom) ~ pov.pred.seq,                                  #plot
response values
```

```
      col = rev(viridis(n_levs))[i],                                       #color
blind safe
      lwd = 2
      )
}

with(subset(df, year == year_seq[j]),                                      #plot
points for focal year
     points(homicide_rate ~ poverty_prop,
            col = adjustcolor("lightgrey", alpha = 0.3),
            pch = 19
            )
     )

legend("topleft",                                                          #legen
d for each Gini quantile
       bty = "n",
       legend = format(round(ine.pred.lev, 2), 2),
       col = rev(viridis(n_levs)),
       lwd = 2,
       cex = 0.7,
       title = "Inequality")

mtext(side = 3, adj = 0, line = 0, paste(year_seq[j]), cex=0.65)

#mtext(side = 3, adj = 0, line = 0.5, "a)")

if(year_seq[j]=="2020")                                                    #add a
xis on bottom plot
  {axis(side = 1, at = seq(0, 0.25, by = 0.05))}
if(year_seq[j]=="2020")                                                    #add a
xis label on bottom plot
  {mtext(side = 1, line = 2, text = "Proportion in Poverty", cex=0.65)}

box()

#right panel

plot(NA,                                                                   #blank
plot
     xlim = c(min(df$gini_index), max(df$gini_index)),                     #range
of Gini index values
     ylim = c(0 , max(df$homicide_rate)+5),                                #range
of homicide values + 5
     xlab = "",
     xaxt = "n",
     ylab = "",
     yaxt = "n"
     )
```

```
#add lines
for(i in 1:n_levs){                                                       #loop
to plot for each quantile
pov.pred.lev <- with(subset(df, year == year_seq[j]),
                            quantile(poverty_prop)                        #quant
ile levels of observed poverty in focal year
                     )


ine.pred.seq <- with(subset(df, year == year_seq[j]),                     #seque
nce to predict across range of poverty for focal year
                     seq(min(gini_index),
                         max(gini_index),
                         length.out = n.int)
                     )

pred.hom.ine <- data.frame(poverty_prop = rep(pov.pred.lev[i], n.int), #new d
ata for each quantile of poverty
                           gini_index = ine.pred.seq,                     #acros
s range of Gini
                           year = rep(year_seq[j], n.int)                 #for f
ocal year
                           )

pred.hom <- predict(b.mod, newdata = pred.hom.ine)                        #predi
ct values

lines(i.link(pred.hom) ~ ine.pred.seq,                                    #plot
response values
      col = rev(viridis(n_levs))[i],                                      #color
blind safe
      lwd = 2
      )
}

with(subset(df, year == year_seq[j]),                                     #plot
points for focal year
     points(homicide_rate ~ gini_index,
            col = adjustcolor("lightgrey", alpha = 0.3),
            pch = 19
            )
     )

legend("topleft",                                                         #legen
d for each poverty quantile
       bty = "n",
       legend = format(round(pov.pred.lev, 2), 2),
       col = rev(viridis(n_levs)),
```

```
        lwd = 2,
        cex = 0.7,
        title = "Poverty")

#mtext(side = 3, adj = 0, line = 0.5, "b)")

if(year_seq[j]=="2020"){axis(side = 1,                             #add ax
is on bottom plot
                             at = seq(0.4, 0.6, by = 0.05))}
if(year_seq[j]=="2020"){mtext(side = 1,                            #add ax
is label on bottom plot
                              line = 2,
                              text = "Inequality (Gini Index)", cex=0.65)}

box()

}
```

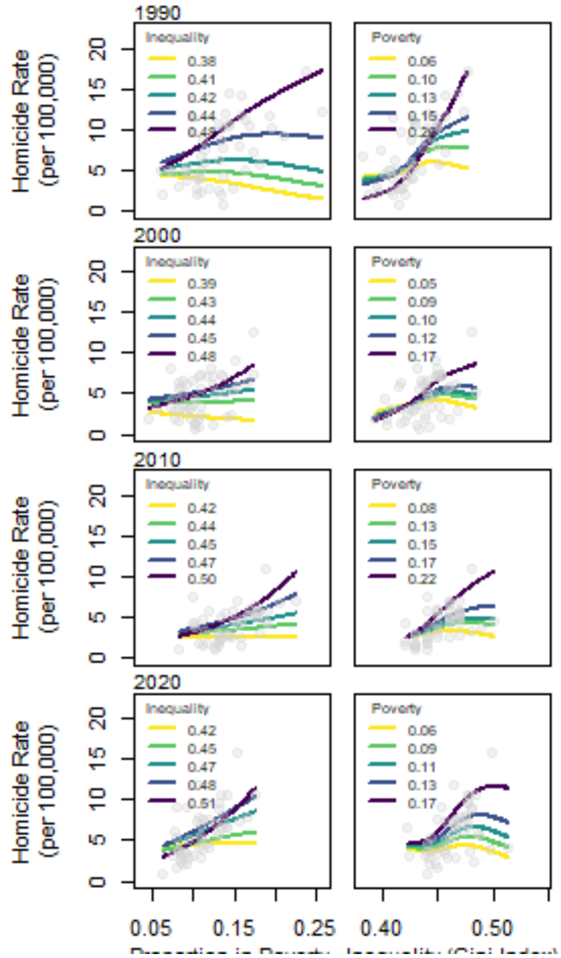


```
#dev.off()
```

Legend for Dataset S1

- year = record year
- state = reporting state
- homicide_rate = total number of homicides per 100,000 people
- gini_index = a measure of income distribution from 0 - 1
- poverty_prop = proportion of people with income under the poverty level

SI References

1. R Core Team (2021). R: A language and environment for statistical computing. R Foundation for Statistical Computing, Vienna, Austria. URL https://www.R-project.org/.

2. Wood, S.N. (2003) Thin-plate regression splines. *Journal of the Royal Statistical Society (B)* 65(1):95-114.

3. Wood, S.N. (2008). Fast stable direct fitting and smoothness selection for generalized additive models. *Journal of the Royal Statistical Society (B)* 70(3):495-518

4. Wood, S.N. (2011) Fast stable restricted maximum likelihood and marginal likelihood estimation of semiparametric generalized linear models. *Journal of the Royal Statistical Society* (B) 73(1):3-36.

5. Wood, S.N. (2017) *Generalized Additive Models: An Introduction with R* (2nd edition). Chapman and Hall/CRC.

6. Wood S.N., N. Pya and B. Saefken (2016) Smoothing parameter and model selection for general smooth models (with discussion). *Journal of the American Statistical Association* 111:1548-1575.

7. Garnier, S. Ross, N. Rudis, R. Camargo, A.P., Sciaini, M. and Scherer, C. (2021). Rvision - Colorblind-Friendly Color Maps for R. R package version 0.6.2.

8. Rudis, B., N. Ross and S. Garnier (2021). Introduction to the viridis color maps. URL https://cran.r-project.org/web/packages/viridis/vignettes/intro-to-viridis.html.

9. Dormann, C.F., Elith, J., Bacher, S., Buchmann, C., Carl, G., Carré, G., Marquéz, J.R.G., Gruber, B., Lafourcade, B., Leitão, P.J. and Münkemüller, T., (2013). Collinearity: a review of methods to deal with it and a simulation study evaluating their performance. *Ecography*, 36(1), pp.27-46.
